# Queensland Consumers’ Awareness and Understanding of Clinical Genetics Services

**DOI:** 10.3389/fgene.2020.537743

**Published:** 2020-10-15

**Authors:** Courtney K. Wallingford, Katrina Cutler, Satrio Nindyo Istiko, Lindsay F. Fowles, Rachel Lamb, Jessica Bean, Louise Healy, Gary Hondow, Gregory Pratt, Miranda E. Vidgen, Nicola Waddell, Erin Evans, David Bunker, Aideen M. McInerney-Leo

**Affiliations:** ^1^Dermatology Research Centre, The University of Queensland Diamantina Institute, University of Queensland, Brisbane, QLD, Australia; ^2^Graduate School of Health, University of Technology Sydney, Sydney, NSW, Australia; ^3^Queensland Genomics, Brisbane, QLD, Australia; ^4^Genetic Health Queensland, Royal Brisbane and Women’s Hospital, Brisbane, QLD, Australia; ^5^QIMR Berghofer Medical Research Institute, Brisbane, QLD, Australia

**Keywords:** health consumers, genetics, genomics, awareness, attitudes

## Abstract

As genetic testing becomes increasingly utilized in health care, consumer awareness and understanding is critical. Both are reported to be low in Australia, though there are limited studies to date. A consumer survey assessed perceived knowledge, awareness and attitudes toward genetic medicine, prior to consumers’ genomics forums in Queensland in 2018 and 2019. Data was analyzed using *t*-test and Mann-Whitney *U* tests analysis to detect any associations between sociodemographic factors and familiarity or attitudes. This highly educated and experienced health consumer cohort reported they were significantly more familiar with the healthcare system generally than genetic medicine specifically (*p* < 0.0001). Consumers perceived that genetic testing would be significantly more important in the future than it is currently (*p* < 0.00001). Consumers agreed that genetic testing should be promoted (91.4%), made available (100%), better funded (94.2%), and offered to all pregnant women (81.6%). The preferred learning modality about genetics was internet sites (62.7%) followed by talks/presentations (30.8%). Benefits of genetic testing, reported in qualitative responses, included the potential for additional information to promote personal control and improve healthcare. Perceived concerns included ethical implications (including privacy and discrimination), and current limitations of science, knowledge and/or practice. This study demonstrates that even knowledgeable consumers have little familiarity with genetic medicine but are optimistic about its potential benefits. Ethical concerns, particularly concerns regarding genetic discrimination should inform legislation and policy. Consumers are supportive of online resources in increasing genomic literacy.

## Introduction

The demand and utilization of genetic and more recently genomic testing continues to increase (Khan and Mittelman, 2018), yet all stakeholders in healthcare report low levels of awareness, comfort and confidence. For physicians, genetic education and training is limited (Harris et al., 2006; Harding et al., 2019). Internationally, non-genetic health professionals admit to having insufficient knowledge of genetics, and the role of clinical genetics services (Houwink et al., 2011; Crellin et al., 2019; Harding et al., 2019). Similarly, the general public report low awareness of genetic risk factors, and knowledge regarding how to access genetic services in healthcare (Smerecnik et al., 2008; Hann et al., 2017).

Previous research has focused on attitudes, awareness and knowledge of health consumers. Throughout various countries, reported genetic literacy is low and consumers are typically uncertain about how it applies to healthcare (Harris et al., 2006; Smerecnik et al., 2008; Hann et al., 2017). Contrary to reports of low genetic literacy, consumers express interest in understanding hereditary risk and how to manage personal genetic risk factors, but lack comprehension in genetic risk perception, often basing it on family history and personal experience (Henneman et al., 2003; Smerecnik et al., 2008; Taylor, 2011). Knowledge in genetics, especially pertaining to healthcare strongly depends upon sociodemographic factors, where those with lower levels of education, older age, or of ethnic minority, tend to have lower knowledge and understanding of genetics, and are more likely to adopt a deterministic view around genetic test results (Morren et al., 2007; Smerecnik et al., 2008; Kaphingst et al., 2012; Rubinsak et al., 2019). Notably, attitudes are predominantly positive toward genetic testing, and higher levels of genetic knowledge are associated with more positive views (Henneman et al., 2003; Morren et al., 2007; Meisel et al., 2016).

Australia has a publicly funded health system which supports access to genetic and genomic testing, when clinically indicated (Australian Law Reform Commission [ALRC], 2010). Despite its availability there are limited studies about consumer knowledge, awareness, and attitudes toward genetic testing in Australia. Previous studies reveal health consumers in Australia are interested in genetic testing but fear genetic discrimination (Molster et al., 2009). They show lower knowledge and negative attitudes toward genetic testing are related to lower education levels, lower household income, and older age (Wilde et al., 2010). Lastly, they indicate a need for more genetic education in primary healthcare providers due to the low uptake of clinical genetic services (Metcalfe et al., 2002).

This study involved the administration of a survey assessing consumers’ awareness and attitudes toward genetics in healthcare prior to a genomics educational intervention in the form of a presentation during the health consumer forum in Queensland in both 2018 and 2019.

## Materials and Methods

### Ethics

This survey was initially administered as a marketing tool to shape the content of future educational sessions. An ethics application to report the findings in the literature was approved by the University of Queensland Human Research Ethics Committee (UQ #2019002633) with the stipulation that quotes be paraphrased.

### Participants

All delegates attending the Queensland Genomics panel presentation on genomics in medicine, at the Health Consumer Queensland annual forum in 2018 (Brisbane) and 2019 (Cairns) were invited to complete the survey. Any individuals present both years (2018 and 2019) noted this on their 2019 survey and only their first response was included in the analysis.

### Data Collection

Surveys were administered and collected prior to the genomics education session. In 2018 the 23-item survey included: 5-items assessing demographic information (age, gender, marital status, education level and Aboriginal/Torres Strait Islander identity); 5-items assessing familiarity with genetic diseases and testing (Yes/No) (3 of which were previously validated by Henneman et al., 2003); two items rating (scale 1–10) their familiarity with the healthcare system generally and genomic medicine specifically; two items rating (scale 1–10) their perceptions regarding the importance of genetics in healthcare currently and in the future; a rank ordered question assessing learning modality preferences; four-items assessing attitudes (agree/disagree) toward the availability of genetic testing; one item assessing whether they could knew how to find a genetics service; two open-ended fields evaluating perceived benefits and concerns with genomic testing and one final open-ended field for additional comments (see Table 1 for specific wording). An additional question was added in the 2019 survey (“Did you attend the HCQ Forum in Brisbane in 2018?”) to allow for the removal of duplicates. See Supplementary Material for full questionnaire.

**TABLE 1:** Summary of consumer survey responses from 59 participants evaluating genomic familiarity, awareness, and beliefs, preferences for learning modalities, and attitudes toward genetic testing.

Topic	Questions/Items	Yes, N (%)
Familiarity (*Yes/No*)	Do you know anyone with a genetic disorder (yourself, in your family or neighborhood)?	56 (71.4)
	Have you heard or read about genetic testing before attending this forum?	59 (81.4)
	Did you, your partner or your children ever have a genetic test?	58 (17.2)
	Have you heard of genomic medicine before attending this forum	59 (66.1)
	If you needed to, would you know how to find genetic services in Queensland?	58 (40.4)
		Average rating* (Range)
Beliefs	How much does genetic testing affect healthcare in Queensland today?	4.97 (1–10)
	How much will genetic testing affect healthcare in Queensland in the future?	**7.97** (1–10)
Awareness	How familiar are you with the healthcare system in Queensland?	6.64 (1–10)
	How familiar are you with genomic medicine?	**2.97** (1–8)
		**Agree (%)**
Attitudes (*Agree/Disagree*)	The use of genetic testing should be promoted?	48 (94.1)
	Genetic testing should be available for those who want to use them?	52 (100)
	More money should be available for the development of genetic tests?	49 (94.2)
	Genetic tests should be offered to all pregnant women?	49 (81.6)
		Average ranking**
Preferences for learning about genetics	Internet sites	1.88
	Talks and presentations	2.40
	Discussions with healthcare providers	2.84
	Videos	3.02
	Printed materials	3.28

*On a scale of 1–10 (with 1 being the lowest). **Rank them from 1 to 5, with 1 being the most useful and 5 being the least useful. Bolding indicates significance.

The survey that was administered used the terms “genetic” and “genomic” interchangeably. We use “genetic” to encompass both terms throughout this article.

### Data Analysis

All responses on 1–10 scales given as a range were converted to averages (i.e., “3–4” was changed to “3.5”). Ranks on scales of “1–5” which were reported as >5 were adjusted to “5.” Participants choosing both “agree” and “disagree” were categorized as “neither agree nor disagree.”

Descriptive statistics were performed to summarize and describe the characteristics of the data. Student’s *t*-tests were performed to test to detect any differences between means in normally distributed data (familiarity with healthcare and sociodemographic factors) and Mann-Whitney *U* tests were used to detect differences in means in datasets which were not normally distributed (Importance of genetics in healthcare now and in the future, and learning preferences).

### Thematic Analysis

All quotes in open-ended fields were transcribed verbatim and analyzed thematically to identify perceived benefits and concerns regarding genetic testing. Codes were categorized to identify overarching themes. Codes were reconciled between two researchers (CW and AM). Themes were reported as well as the frequency with which they appeared in the text. Illustrative quotes were paraphrased to preserve consumer privacy.

## Results

A total of 66 individuals participated in the survey in 2018 (*n* = 51) and 2019 (*n* = 15). Seven individuals in the 2019 survey reported having attended the Queensland Genomics presentation for the Health Consumer Queensland annual forum in 2018, and were therefore removed from statistical analyses, resulting in 59 unique individuals.

### Demographics

The majority of participants were female [*n* = 41 (69.5%)], 16 were males (27.1%), and 2 were not specified (3.4%). Ages of participants ranged from 26 to 80 years of age with the average age being 47.3 years. This cohort was highly educated with 50 participants (84.7%) having some form of tertiary education and 28 participants (47.5%) having undertaken graduate education.

### Familiarity With Genetics and Healthcare

Of the participants who completed each question, 40/56 (71.4%) reported knowing someone with a genetic disorder personally, 11/59 (18.6%) had never heard of genetic testing before attending the forum, and 10/58 (17.2%) reported personal experience with genetic testing. Finally, 34/58 (59.6%) did not know how to find genetic services in Queensland.

The average self-reported familiarity with the healthcare system in Queensland was 6.64 (range 1–10), and the median was 7. The average genetic medicine familiarity score was 2.97 (range 1–8) with a median of 1. *T*-tests found these scores to be significantly different (refer to Table 1) (*t* = 8.71, *p* < 0.0001). There were no correlations between these scores and age or education.

### Beliefs About Genetic Medicine

Participants perceived that genetic testing moderately affected healthcare in Queensland today with a mean of 4.97 (range 1–10) and median of 5 (Table 1). Participants perceived that genetic testing would be significantly more relevant to healthcare in Queensland in the future than it is presently with a mean of 7.97 (range 1–10) (Table 1) (*z*-score −4.634 *p* < 0.00001). There were no correlations between these scores and age or education for either time point.

### Attitudes Toward Genetic Testing

Of the participants who provided a response, 48/51 participants (94.1%) agreed that the use of genetic testing should be promoted with 52/52 (100%) agreeing that genetic testing should be available for those who want to use them, 49/52 (94.2%) agreeing that there should be more funding and 40/49 (81.6%) agreeing that genetic tests should be offered to all pregnant women (Table 1). There were no demographic predictors of these ratings.

### Preferences for Learning

Participants were asked to rank modalities from 1 to 5 with 1 being the highest. The preferred learning modality about genetics was internet sites 37/59 (62.7%) followed by talks/presentations 16/52 (30.8%). The average ranked score for each modality was calculated (Table 1). Internet sites received an average score of 1.88, talks and presentations 2.4, discussions with healthcare providers 2.84, Videos 3.02 and printed materials 3.28. Mann-Whitney *U-*tests (Table 2) found that internet sites were significantly preferable to all other learning modalities including printed materials (*p* < 0.00001), videos (*p* = 0.00001), discussions with health care providers (*p* = 0.001) and talks and presentations (*p* = 0.019). Talks and presentations were significantly preferable to printed material (*p* = 0.003) and videos (*p* = 0.003) but not discussions with healthcare providers (*p* = 0.265). Mann-Whitney *U* test found that individuals under 45 years of age (*n* = 25) also preferred internet sites above videos (*p* = 0.001), discussions with healthcare providers (*p* = 0.032), and printed material (*p* = 0.00005), but there was no differences between their preference for internet resources as compared to talks and presentations (*p* = 0.172). Those over the age 45 years (*n* = 29) preferred internet sites to videos (*p* = 0.031), discussions with physicians (*p* = 0.033) and printed materials (*p* = 0.028), but there were no other significant preferences (Table 2).

**TABLE 2:** Mann-Whitney *U-*test analysis evaluating preferences of specific learning modalities for genetic education.

Learning modalities	<45 years (*N* = 25)		≥45 years (*N* = 29)		Total cohort (*N* = 54)	
	Average ranking	*P*-value	Average ranking	*P*-value	Average ranking	*P*-value
Internet vs. Videos	1.65 vs. 3.18	**0.0001**	2.16 vs. 2.95	**0.031**	1.88 vs. 3.02	**<0.00001**
Internet vs. Talks/Presentations	1.65 vs. 2.23	0.172	2.16 vs. 2.45	0.105	1.88 vs. 2.40	**0.019**
Internet vs. Discussions with physician	1.65 vs. 2.82	**0.032**	2.16 vs. 2.75	**0.033**	1.88 vs. 2.83	**0.001**
Internet vs. Printed material	1.65 vs. 3.62	**0.00005**	2.16 vs. 3.05	**0.028**	1.88 vs. 3.28	**0.00001**
Printed material vs. Videos	3.62 vs. 3.18	0.590	3.05 vs. 2.95	0.516	3.28 vs. 3.02	0.641
Printed material vs. Talks/Presentations	3.62 vs. 2.23	**0.00055**	3.05 vs. 2.45	0.252	3.28 vs. 2.40	**0.003**
Printed material vs. Discussions with physician	3.62 vs. 2.82	0.**035**	3.05 vs. 2.75	0.451	3.28 vs. 2.83	0.126
Videos vs. Talks/Presentations	3.18 vs. 2.23	**0.002**	2.95 vs. 2.45	0.367	3.02 vs. 2.40	**0.003**
Videos vs. Discussions with physician	3.18 vs. 2.82	0.095	2.95 vs. 2.75	0.640	3.02 vs. 2.83	0.2083
Talks/Presentations vs. Discussions with physician	2.23 vs. 2.82	0.297	2.45 vs. 2.75	0.627	2.40 vs. 2.83	0.265

*Bolding denotes significance.*

### Perceived Benefits of Genetics

Forty-seven written responses identified two main categories of benefits (Table 3). More than half of the responses (28/47) mentioned that genetic testing provided information that promoted control and approximately one third (19/47) thought that genetic testing could improve health care.

**TABLE 3:** Qualitative responses in open-ended questions regarding perceived concerns and benefits of genetic testing (*n* = 47).

Themes	Paraphrased quotes	N (%)
**Concerns**		
Ethical implications	• I am worried about my privacy being invaded. • Genetic discrimination and inequality. • You might be influenced to make decisions around continuing pregnancies.	32 *(68.1%)*
Limitations of service/science/information	• Genetic tests might be inaccurate. • You might discover you have a condition for which there is no treatment. • Fear of uncertainty regarding the implications of genetic test results.	12 *(25.5%)*
**Benefits**		
Provides information promoting control	• Information means that patients can make informed decisions. • Genetic testing can give you information that allows you to consider family planning. • Being aware of genetic risk factors means you can make decisions about your health, and plan for the future.	28 *(59.6%)*
Improve healthcare	• Genetic testing might allow for more personalized health care. • We might develop better treatments by increasing our understanding of conditions using genetic testing. • Early detection of a condition may lead to earlier intervention.	19 *(40.4%)*

Forty-seven individuals provided responses describing their concerns with genetic testing which could be broadly categorized into two themes, namely ethical considerations (*n* = 32) and knowledge limitations inhibiting interpretation and utilization (*n* = 12).

## Discussion

Health consumers in this study were highly educated and over two thirds personally knew an individual with a genetic condition. Participants rated their knowledge of the healthcare system in Queensland to be relatively high but their perceived familiarity with genetic medicine was significantly lower. The majority of respondents did not know how to find genetic services in Queensland. There was overwhelming support for integration of genetic testing into healthcare with the majority believing it to have a greater impact in the future than it did at present. Qualitative comments on perceived concerns included possible ethical implications and the current limitations of science and technology. Perceived benefits centered on the potential for genetic information to increase control and to improve healthcare. Internet sites and talks or presentations were the preferred learning modalities for genetic education.

The majority of our cohort personally knew someone with a genetic condition. This may reflect the fact that cumulatively rare disorders affect 6–10% of the world’s population (Zurynski et al., 2008). However, it is likely that this represents a study bias. This population would have an increased *a priori* chance of encountering individuals with rare disorders given that they identified as health consumers who, by definition, frequently attend healthcare centers. Furthermore, the fact that they elected to attend a session on genetics suggests that they had a vested interest in the topic.

Our cohort reported moderate familiarity with the healthcare system in Queensland, which was expected, given that they were attending a health consumers’ forum. However, a previous Australian study found an inverse relationship between consumers’ perceived familiarity with healthcare systems and experience accessing healthcare, suggesting that those who regularly attended healthcare settings found the experience to be more complex than they might have anticipated (Mather et al., 2018). The apparent lack of familiarity with genetic medicine is consistent with low knowledge of medical specialties in Australian consumers (Gianduzzo et al., 2016) and low genetic literacy in the general public worldwide (Smerecnik et al., 2008). The low levels of familiarity are unexpected in this cohort, given their health consumer roles and the fact that they are highly educated. However, previous research has shown that genetic literacy is low, even amongst well-educated populations (Chapman et al., 2019).

The use of genetic testing in medicine will continue to rise (Whitworth et al., 2017; Savard et al., 2019) and it is predicted that 60 million patients will have had whole genome sequencing by 2025 (Birney et al., 2017). It is already being mainstreamed in specific cancer specialties e.g., breast and ovarian cancer, to inform management decisions (Previs et al., 2016; Kentwell et al., 2017). Our findings reflect consumer recognition and support for these trends, with the vast majority of participants believing that genetic testing should be funded and made accessible to the public. This presents a challenge to the healthcare system as it continues to manage the costs and increasing complexity of delivery. It is therefore essential that genetic medicine is used efficiently and in the most clinically relevant settings in order to provide and maintain accessible and high-quality healthcare for all health consumers. Dombrádi et al. (2019) describes this process as a shift to value-based care, and argues that is particularly important with genomic medicine. Participants in this study anticipated that genetic testing would play a significantly larger role in health care in the future than it does currently. This aligns with previous reports that, internationally, consumers have high expectations for the potential of genetics to positively impact on healthcare in the future (Hall et al., 2015; Hayeems et al., 2015; Roberts et al., 2018).

Genetic specialists are increasingly in demand, yet the supply is relatively limited (Heald et al., 2016). With over 75,000 genetic tests available to the public (Phillips et al., 2018), and new tests being developed regularly, consumer demand for informational resources will increase further in the future. Educating consumers about genetics enables them to make informed decisions about their health, and promotes better healthcare (Kaphingst et al., 2012). We found that the most preferred modalities of genetic education in this cohort were internet sites, followed by talks and presentations. These learning preferences were largely attributable to strong preferences in younger participants (under 45 years of age), though the older cohort also preferred internet sites to either videos, printed materials or discussions with healthcare providers. This is unexpected given reports that the utilization of digital information technologies, to obtain health information, decreases with age (Gordon and Crouch, 2019). Online learning, and talks or presentations are both interactive learning modalities, shown to positively impact on consumers’ knowledge and understanding (Alamantariotou and Zisi, 2010). E-learning tools have been used to effectively increase knowledge and promote informed decision making prior to genetic testing (reviewed by Birch, 2015). Increasingly, there are calls to develop educational resources on the topic of genetics in healthcare (Hann et al., 2017; Krakow et al., 2018) and this study supports this. This is the first Australian study to show consumers’ preference for learning about genomics online, which is consistent with a review showing that the majority of recently developed genomic educational resources for the public are digital (Whitley et al., 2020).

Consumers’ concerns regarding genetic testing included possible ethical implications. Specifically, and in keeping with previous research findings, participants expressed concern regarding the potential for genetic discrimination (Wilde et al., 2010), data sharing (Hann et al., 2017), breaches of privacy (Deans et al., 2015), eugenics (Webborn et al., 2015), and the current limitations in technology and knowledge affecting our ability to appropriately interpret complex results (Ravitsky et al., 2017). Concerns of genetic discrimination are particularly warranted with regards to how genetic information is used in determining eligibility and premium costs for certain insurance policies in Australia (Barlow-Stewart et al., 2009; Keogh and Otlowski, 2013; Tiller et al., 2020). This deters health consumers from having genetic testing in both clinical and research settings, resulting in less informed health consumers, and barriers to medical research (Newson et al., 2017; Tiller et al., 2020). A recent study showed that in Australasia, the majority of genetic health professionals felt there was not enough legislation to protect clients from genetic discrimination (Tiller et al., 2018). It is important that the concerns of health consumers are addressed, not only in pre-test education and counseling sessions, but at a government policy and legislative level to ensure the long-term protection for health consumers, and to promote medical progress.

Perceived benefits of genetic testing included results providing consumers with information, which could promote control over healthcare decisions and the potential to improve healthcare. Consistent with the literature, participants reported that genetic testing facilitated planning and preparing for the future (Hamilton et al., 2016; Hann et al., 2017), and the opportunity to engage in preventative interventions (Bloss et al., 2013; Hamilton et al., 2016; Roberts et al., 2017). Additional benefits included the potential for personalized medicine, and the identification of novel treatment and cures (Dolled-Filhart et al., 2012; Hann et al., 2017).

### Limitations

Limitations of this study include a limited sample size and a highly educated and self-selected cohort. As surveys were administered at the Health Consumer Queensland Forum, this sample is likely to be more highly motivated and educated than typical healthcare consumers. Therefore, the findings are not generalizable to all health consumer groups. Nonetheless, the findings do provide insight into the attitudes toward and awareness of genetics in healthcare in Queensland.

## Conclusion

This study demonstrates that even highly educated healthcare consumers, who are familiar with their local healthcare systems, are significantly less familiar with clinical genetic services, and how to access them. Consumers recognized the positive potential of genetics and felt it would play a more significant role in healthcare in the future than it does today. Online educational interventions were consumers’ preferred medium for learning about genomics.

## Data Availability Statement

The datasets generated for this study are available on request to the corresponding author.

## Ethics Statement

The studies involving human participants were reviewed and approved by the University of Queensland Human Research Ethics Committee (UQ #2019002633). Written informed consent for participation was not required for this study in accordance with the national legislation and the institutional requirements.

## Author Contributions

AM-L, CW, DB, KC, and EE established the study and applied for ethics. AM-L, LF, SI, and RL developed the survey which was administered by KC. Presenters for the Queensland Genomics panel at the Health Consumer Queensland forum were KC, AM-L, NW, GH, DB, and EE (2018), and EE, SI, GH, and LF (2019). CW and AM-L co-wrote the manuscript. All authors critically reviewed the manuscript.

## Conflict of Interest

NW was the co-founder, minor equity holder, and board member of genomiQa. The remaining authors declare that the research was conducted in the absence of any commercial or financial relationships that could be construed as a potential conflict of interest.
