# Supplementary material for: Queensland Consumers’ Awareness and Understanding of Clinical Genetics Services
Source: Front Genet. 2020 Oct 15;11:537743. doi: 10.3389/fgene.2020.537743 (PMC7593610; doi:10.3389/fgene.2020.537743)
Supplement: Supplementary file 1 [file Table_1.DOCX]

Supplemental Data: Questionnaire

Queensland Genomics Session: Bringing genomics to the community – what do you want to know?

Audience Survey

# Health Consumers Queensland Forum

**Wednesday 5 June 2019 | Hilton Cairns, 34 Esplanade, Cairns**

**Demographic information:**

Age:

Gender:

Marital Status:

Education level:

Do you identify as Aboriginal or Torres Strait Islander? **YES/NO** (please circle)

Did you attend the Health Consumers Queensland Forum in Brisbane 2018 **YES/NO** (please circle)

If **YES**, did you complete the questionnaire at the Queensland Genomics Session? **YES/NO/DON’T REMEMBER**

(please circle)

**Survey questions:**

1. *Your familiarity with genetic diseases and tests:*
   1. Do you know anyone with a genetic disorder (yourself, in your family or neighbourhood)?

**YES/NO** (please circle)

- 1. Have you heard or read about genetic testing before attending this forum?

**YES/NO** (please circle)

- 1. Did you, your partner or your children ever have a genetic test?

**YES/NO** (please circle)

- 1. Have you heard of genomic medicine before attending this forum? **YES/NO** (please circle)

1. *Your awareness about genomics relative to your awareness about other health services:*
   1. On a scale of 1 to 10 (with 1 being the lowest), how familiar are you with the healthcare system in Queensland?

1

10

- 1. On a scale of 1 to 10 (with 1 being the lowest), how familiar are you with genomic medicine?

1

10

- 1. If you needed to, would you know how to find genetic services in Queensland?

**YES/NO** (please circle)

1. *Your beliefs regarding the significance of genomics in healthcare:*
   1. On a scale of 1 to 10 (with 1 being the lowest), how much does genetic testing affect healthcare in Queensland today?

1

10

- 1. On a scale of 1 to 10 (with 1 being the lowest), how much will genetic testing affect healthcare in Queensland in the future?

1

10

1. *Your preferences for how you would like to learn about genetics/genomics:*
   1. If you wanted to learn more about genetics, which of the following would be most useful to you? (Rank them from 1 to 5, with 1 being the most useful and 5 being the least useful)

…….. Printed materials

…….. Internet sites

…….. Videos

…….. Talks and presentations

…….. Discussions with healthcare providers

1. *Your attitudes towards the availability of genetic testing:*
   1. The use of genetic testing should be promoted

**Agree/Disagree** (please circle)

- 1. Genetic testing should be available for those who want to use them

**Agree/Disagree** (please circle)

- 1. More money should be available for the development of genetic tests

**Agree/Disagree** (please circle)

- 1. Genetic Tests should be offered to all pregnant women

**Agree/Disagree** (please circle)

1. *In your opinion, what are the main benefits of genomic testing?*

1. *In your opinion, what are the main concerns with genomic testing?*

1. *Additional comments*
